# Guiding principle of reservoir computing based on “small-world” network

**DOI:** 10.1038/s41598-022-21235-y

**Published:** 2022-10-06

**Authors:** Ken-ichi Kitayama

**Affiliations:** 1 National Institute of Information and Communications Technology, Tokyo, 184-8795 Japan; 2 Hamamatsu Photonics K.K., Hamamatsu, 434-8601 Japan

**Keywords:** Mathematics and computing, Optics and photonics

## Abstract

Reservoir computing is a computational framework of recurrent neural networks and is gaining attentions because of its drastically simplified training process. For a given task to solve, however, the methodology has not yet been established how to construct an optimal reservoir. While, “small-world” network has been known to represent networks in real-world such as biological systems and social community. This network is categorized amongst those that are completely regular and totally disordered, and it is characterized by highly-clustered nodes with a short path length. This study aims at providing a guiding principle of systematic synthesis of desired reservoirs by taking advantage of controllable parameters of the small-world network. We will validate the methodology using two different types of benchmark tests—classification task and prediction task.

## Introduction

**Figure 1:**
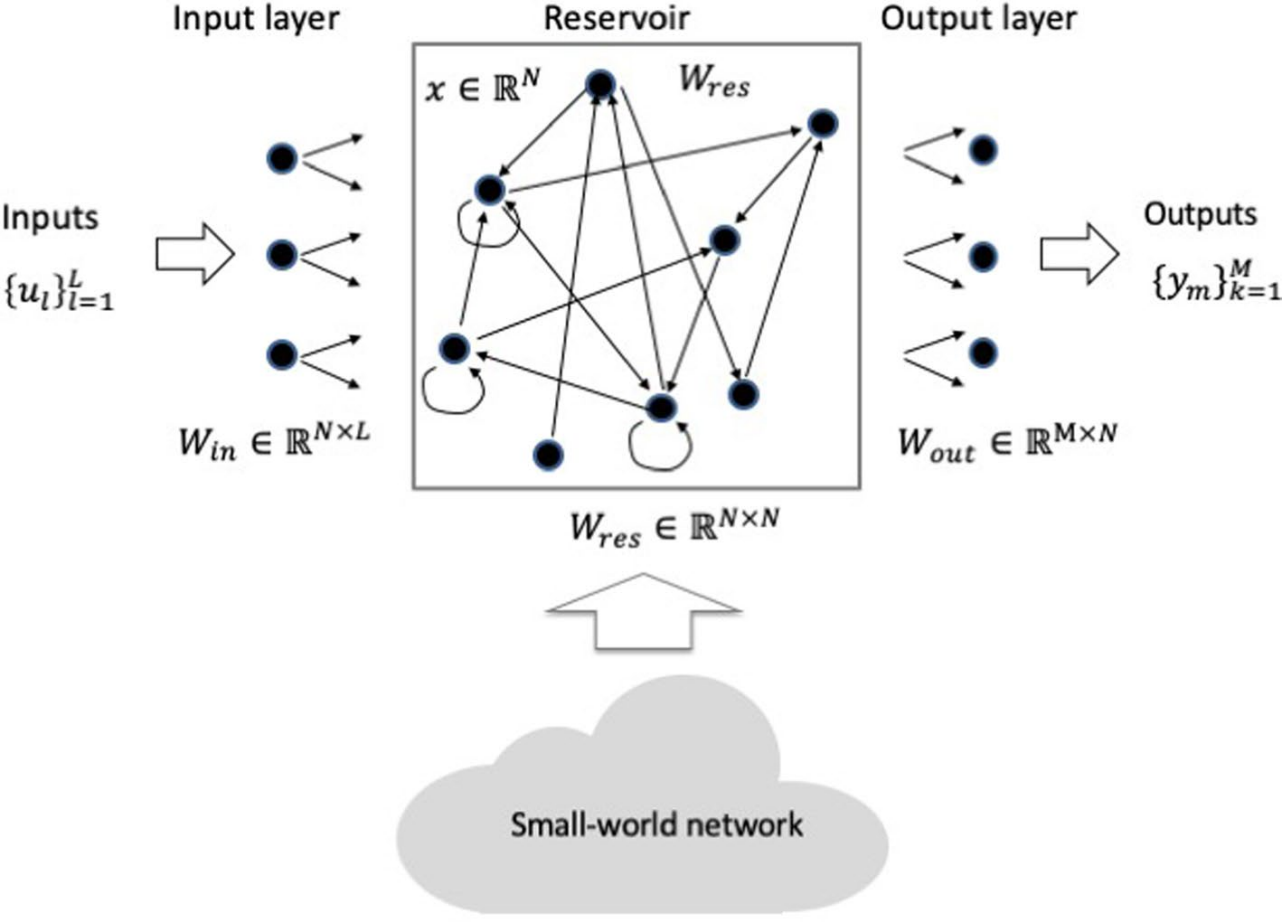
Reservoir computing architecture. Input weight matrix *W*_in_ is a fixed *N* × *L* matrix where *N* is the number of nodes in the reservoir, and *L* is the dimension of the inputs at each time step. Reservoir weight matrix *W*res is a fixed *N* × *N* matrix, which is typically sparse with nonzero elements having an either a symmetrical uniform, discrete bi-valued, or normal distribution centered around zero^13^. Output weight matrix *W*out is a learned $M\times N$ matrix where $M$ is the number of classes of the output data.

Reservoir computing (RC) is a unified computational framework^1,2^, independently proposed recurrent neural network (RNN) models of echo state networks (ESNs)^3,4^ and liquid state machines (LSMs)^5,6^. It is a special class of RNN models, consisting of three layers—an input layer and an output layers and a reservoir between the input and output layers (Fig. 1). The primary difference between the RC and deep learning or multi-layer neural networks is that only the connections between the reservoir and the output layer are trainable, and the training requires much less data than in the deep learning. Owing to an excellent memory capability of the recurrent nature, it can be used in speech recognition and temporal waveform forecast. It has been shown that the RC can be used for the prediction and recognition of temporal and sequential data such as spoken word^5^, time series signals^4,6^, and wireless and optical a channel equalizations^7,8^. In addition, RC can also be used for handwritten digits recognition by transforming the images into temporal signals^9^.

There have been several studies on various reservoir network topologies such as sparsely random network^1,2^ and topologies of swirl^10^ and waterfall^11^. However, for every given task, one has to empirically seek an optimum condition, and these topologies are not sufficiently flexible because there are few adjustable parameters. Hence, a variety of networks has been tested for the reservoir. In a conventional random reservoir, it is treated like a “black box”, which only allows one to specify the density or the sparseness of the weight matrix of the reservoir. The topologies of the swirl and the waterfall are fixed, and there is no variable except for the size, that is, the number of nodes. Therefore, a guiding principle to find an optimal reservoir for a given task is required. The forementioned factors have motivated us toward using “small-world” network^12^ for the reservoir.

The reservoir state vector $x\left(t\right)$ and the output vector $y\left(t\right)$ at time *t* are given by^13^

1 $$x\left(t+1\right)=\left(1-\alpha \right)x\left(t\right)+\alpha f\left({W}_{res}x\left(t\right)+\gamma {W}_{in}u\left(t\right)\right),$$

2 $$y\left(t+1\right)={W}_{out}x(t)$$

where $\alpha \in [\mathrm{0,1}]$ is the leaking rate, $f(\cdot )$ the activation function of node, and $\gamma$ the input gain. When $\alpha$ is equal to zero, the states are totally governed by previous states, while for the case with $\alpha =1$, the next state of the reservoir depends only on the current state and the external input. Their weights are uniquely determined using ${W}_{out}{x}_{tr}\cong y$ by employing the regularized least squares method as^13^

3 $${W}_{out}=y{x}_{tr}^{T}{({x}_{tr}{x}_{tr}^{T}+\lambda {I}_{N\times N})}^{-1}$$

where ${x}_{ty}$ is the training input vector, $\lambda$ the regularization parameter, and $I$ the *N* × *N* identity matrix. Because the training is simple, the computation cost is low.

Various hardware implementations of the RC based on electronic and photonic components have been reported. The hardware could serve as an accelerator at the frontend of digital computers^14^, which is optimized to perform a specific function but does so faster with less power consumption compared to a general-purpose processor. The electronic RC implementations include analog circuits^15^ and VLSIs^16^, while the photonic hardware of RC exploits its parallelism and high-speed operations with a potentially low power consumption^10,11,17–24^. However, a stumbling block is the absence of nonlinear devices on a large scale acting as the activation function of a reservoir node. To address this issue, two types of architecture of photonic RC have been proposed—delay-loop reservoir and spatial reservoir. The delay-loop RC can simplify a complicated network using a single nonlinear node in a loop-back configuration with the time-delayed feedback. The virtual nodes are distributed along the delay line, and the data injection is realized using time multiplexing^17,18^. In the delay-loop reservoir, an electro-optic modulator^19,20^, a semiconductor optical amplifier^11,21^, and a laser diode^22,23^ can be used as the nonlinear node. The nonlinearity is yielded in the optical output against the applied input voltage of the electro-optic modulator, while it appears in the optical output against the optical input of both the semiconductor optical amplifier and the laser diode, subject to the optical feedback. On the other hand, the spatial implementation of RC is basically a spatially-distributed network^23^. This model uses two key components; a spatial light modulator which consists of over a few tens of thousands of pixels that act as the reservoir nodes and a digital micro-mirror device realizing the reconfigurable output weights. Recently, an alternative approach to building larger reservoirs based on the combination of several blocks of small reservoir has been proposed; the model demonstrated enhanced computational capability^24^. As the photonic integrated circuit (PIC) technology is recently making a rapid progress^25,26^, PIC-based hardwares of the RC will be developed in the near future. This study will also serve as a design guideline of the PIC RC.

With regard to the forementioned factors, we will explore a guiding principle for optimizing the reservoir by shedding light on an article of small-world network in 1998^12^. With the aim of achieving a better performance of the RC, we will refer to several preceding works relevant to small-world network^27–29^. In Ref.^27^, a reservoir model, scale-free highly-clustered echo state network (SHESN) having characteristics of both networks of the small-world and the scale-free^30^ has been proposed. SHESN features a spatially hierarchical and distributed topology where the intradomain connections are much denser than those of interdomain ones. In each domain, the small-world network characteristics such as a short path length and a high clustering are preserved, while the power law degree distribution of the scale-free network is embedded. It is numerically shown that time-series prediction capability of the Mackey–Glass (MG) dynamic system is enhanced, compared with a conventional reservoir having random connections. Reference^28^ analyzes three types of network, including the scale-free network and small-work network as well as their mixture and demonstrates enhanced capability in the prediction of two types of time serial signals generated by NARMAX mode. Reference^29^ investigates characteristics of the path length of three types of network for reservoir, including the small-world network, the scale-free network, and the conventional random one, in order to narrow down the search space of the parameter of the reservoir for predicting chaotic signals. In our present work, the reservoir model is solely based on the small-world network. We examine that the dependence of the parameters such as the degree of nodes *k* and the rewiring probability *p* on the computing capability, which has not been studied in details in the preceding works, Refs.^27–29^. We also show that there is a sweet spot of the small-world network, which gives the optimum performance of the RC for two typical tasks of neural networks; classification and regression. For a classification task, we chose the classification of human activities which has not been tested in the preceding articles, while for a regression task, we conducted the prediction of the MG chaotic signals as Refs.^27,29^ did.

**Figure 2:**
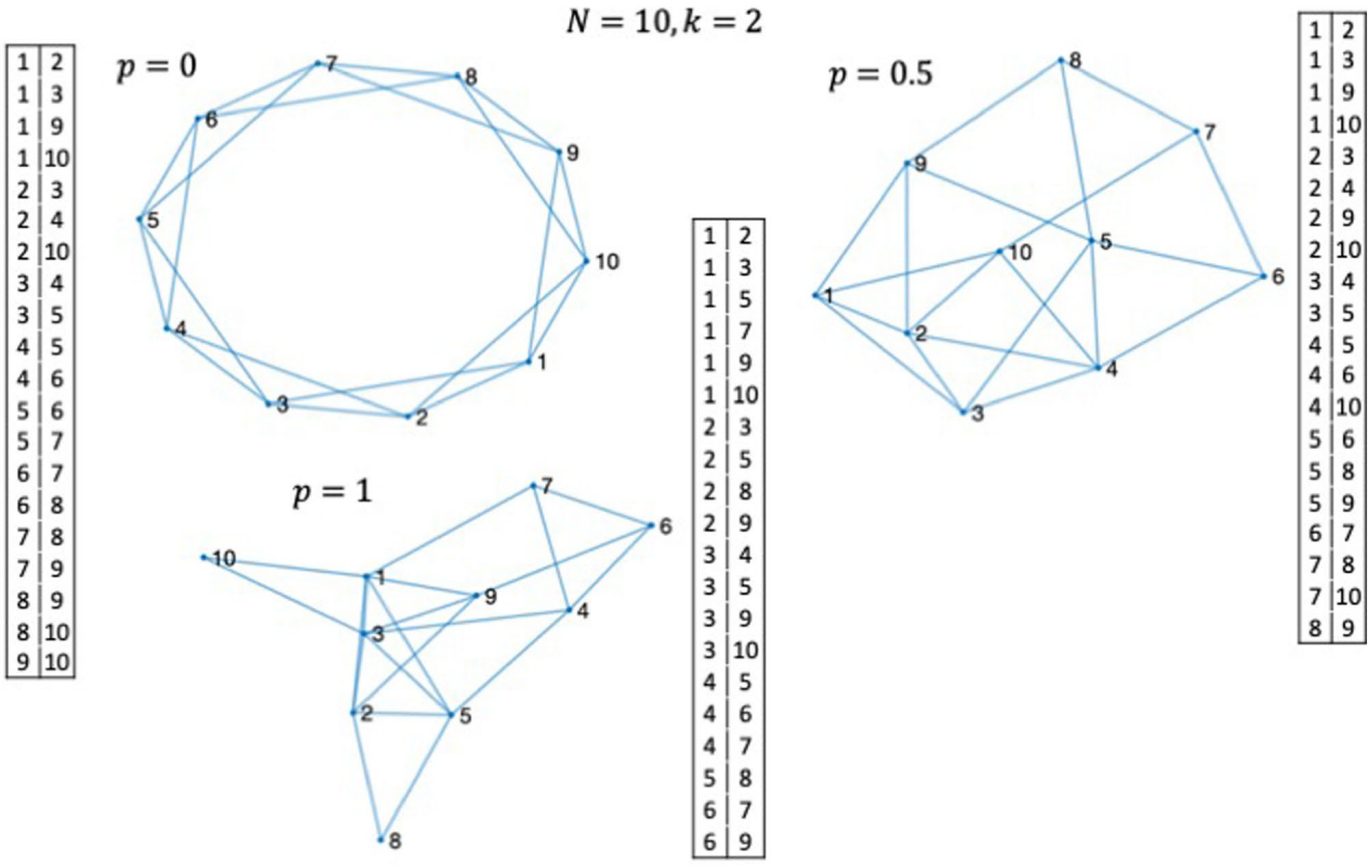
Architectures of 10-node ($$N=10$$) networks with the node degree $$k=2$$; regularly connected ($$p=0$$) on the l.h.s. on the top, modestly disordered ($$p=0.5$$) on the r.h.s. on the top, and totally disordered ($$p=1$$) at the bottom. Each node is connected with four (= 2$$k)$$ neighboring nodes. Each table represents the pairs of nodes for each $$p$$. For example, node#1 is connected with nodes#2, 3, 9, and 10 for the case with $$p=0$$.

The term, small-world network, is derived by an analogy with the small-world phenomenon^31^. It has been shown that the small-world network can well characterize the social and natural phenomena in a real world, including human behavior in social lives, power grid networks, and biological neural networks. We recall a statement related to the ongoing pandemic, presented in the article^12^, “infectious diseases are predicted to spread much more easily and quickly in a small world; the alarming and less obvious point is how few short cuts are needed to make the world small”. The small-world network is based upon the Watts–Strogatz graph, which explores a simple model of network with an arbitrarily-tuned magnitude of disorder by rewiring the links between the nodes. The small-world network is categorized between a regular network ($p=0$) and a completely disordered one ($p=1$), where a small amount of the links between the nodes are rewired to introduce disorder. Here, $p$ is the probability of rewiring the links at random. For the purpose of illustration, three examples of 10-node ($N=10$) network with the node degree $k=2$ are illustrated in Fig. 2, which indicates connections with $2k$ neighboring nodes, including the regularly connected ($p=0$), the modestly disordered ($p=0.5$), and the totally disordered ($p=1$). It is possible to exploit the high flexibility and build up a desired reservoir from scratch. The weight matrix of the reservoir ${W}_{res}$ can be generated from the table of the link connections shown alongside of the graphs (Fig. 2) (see “Methods” section). The link may be either bidirectional or partially bidirectional, and in this study, it is assumed that all the links are bidirectional, thus resulting in a symmetric matrix ${W}_{res}$. There has been another network model referred to as the Erdős–Rényi model^32^, the primary difference between this model and the Watts–Strogatz graph is in the number of parameters, wherein the former contains one parameter, the node degree, while the latter has two parameters, the node degree and the probability of rewiring.

## Results

In the Watts–Strogatz graph, the clustering coefficient of node *i* with the node degree ${k}_{i}$ is defined as^33^

4 $${C}_{i}=\frac{Number \, of \, triangles \, involving \, node i}{Number \, of \, links \, at \, most {k}_{i}\left({k}_{i}-1\right)/2},$$

and the total clustering coefficient of *N*-node network is expressed by

5 $$C=\frac{1}{N}\sum_{i=1}^{N}{C}_{i}.$$

The density of the weight matrix ${W}_{res}$ of the small-world network-based reservoir is calculated by

6 $$Density=\frac{nonzero {\, W}_{res} \, elements }{{N}^{2}}=\frac{N\times 2k}{{N}^{2}}=\frac{2k}{N}.$$

**Figure 3:**
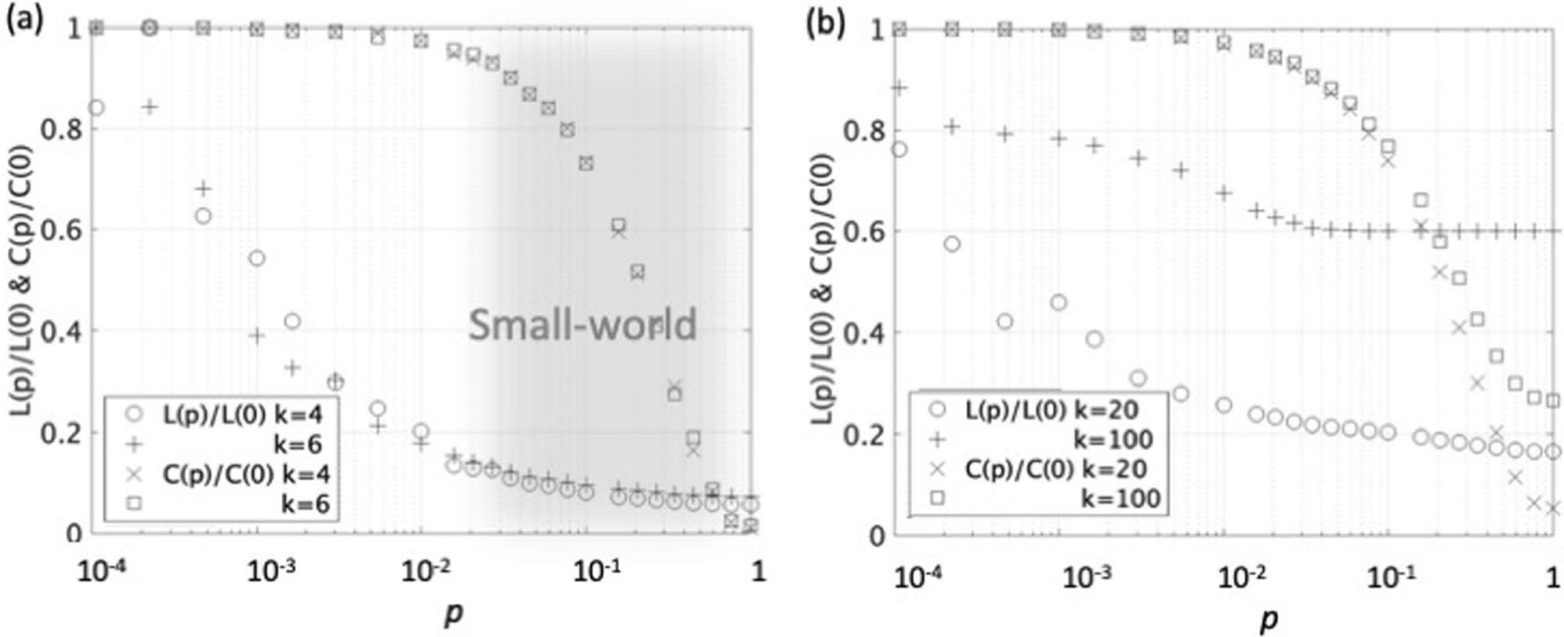
**a** Clustering coefficient $C(p)/C(0)$ and average path length or hop count $L(p)/L(0)$ vs. probability of rewiring *p* for the case with 1000-node ($N=1000$) and the node degrees $k=4$ and 6. Range roughly $p=$ 0.01–0.7 of small-world is indicated by the shaded area. **b** Clustering coefficients $C(p)/C(0)$ and average path lengths $L(p)/L(0)$ vs. probability of rewiring *p* for the case with the node degrees $k=20$ and 100.

The characteristic path length $L(p)$ is calculated as the mean value of distances of the shortest paths between all the nodes. The clustering coefficient $C(p)/C(0)$ and the characteristic path lengths $L(p)/L(0)$ as a function of the probability of rewiring $p$ for the case with 1000-node and the node degrees, $k=4$ and 6 are shown in Fig. 3a. As $p$ increases, the clustering coefficient rapidly decreases beyond $p>0.01$, and the average path length also monotonically decreases. The small-world network is indicated by the shaded area of $p$ = 0.01–0.7, which is characterized highly-clustered with the relatively short path length. As shown in Supple-Fig. 1, there are several clustering hubs for the case with the 1000-node network of $\left(k,p\right)=(4, 0.5)$, which connect with up to 14 ~ 16 nodes are indicated in orange and yellow of the color bar. When the degree $k$ is greater than 20, the small-world characteristic of highly-clustered with the relatively short path length is almost lost (Fig. 3b). Based on this observation, the degree $k$ up to around 20 preserves the characteristic of the small-world.

**Figure 4:**
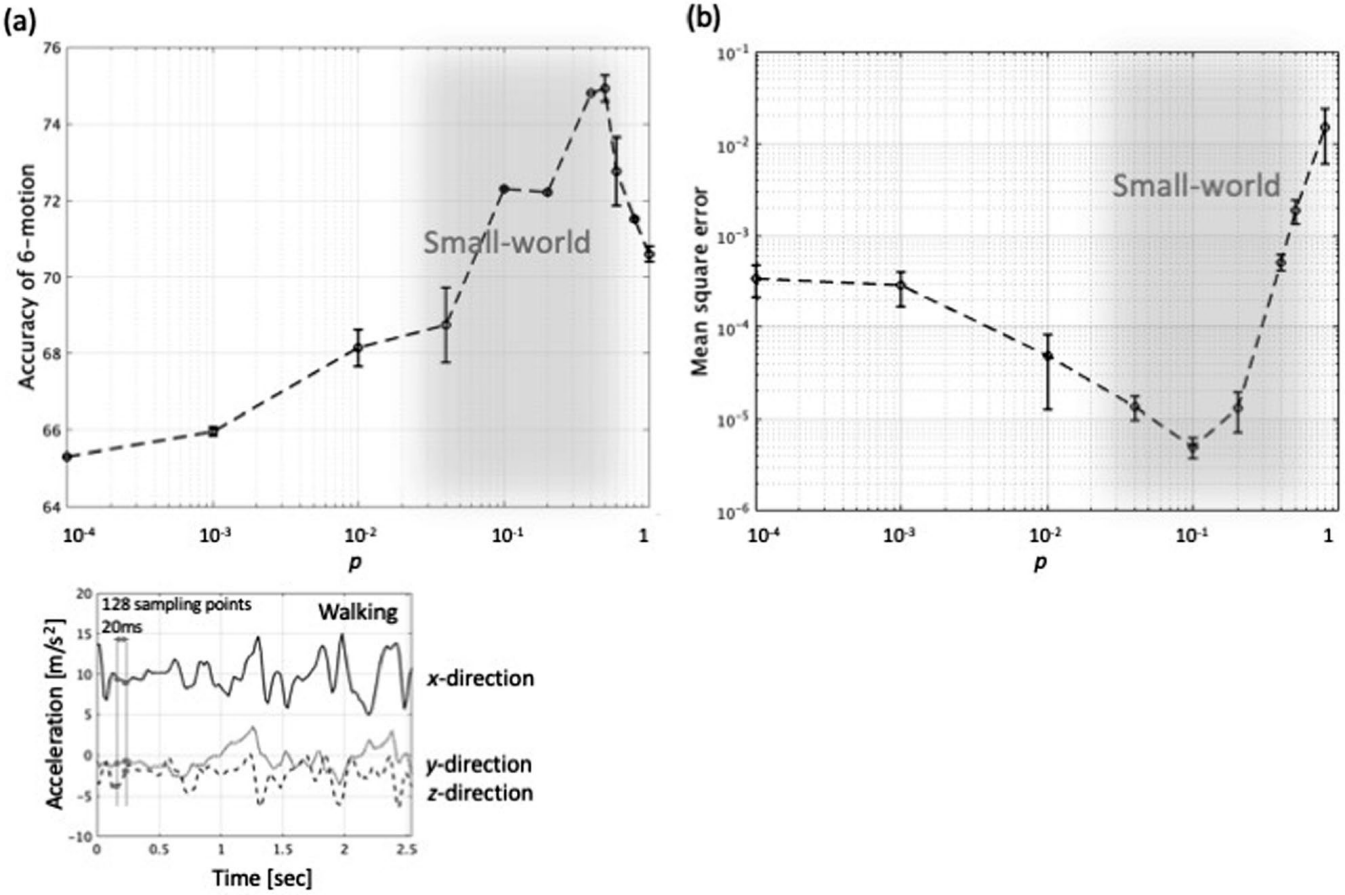
(**a**) Classification accuracy of human 6-activity vs. probability of rewiring $p$ for the case with $N=1000$ and $k=4$. The accuracy is maximized to be 74.9% at $p=0.5$. For an example, the temporal waveforms of accelerations of walking on x-, y, and z-axes are also shown on the bottom. (**b**) Prediction accuracy represented by the mean square error (MSE) of MG chaotic signal vs. $p$ for the case with $N=1000$ and $k=4$. MSE is minimized to be $4.98\times {10}^{-6}$ at $p=0.1$.

**Figure 5:**
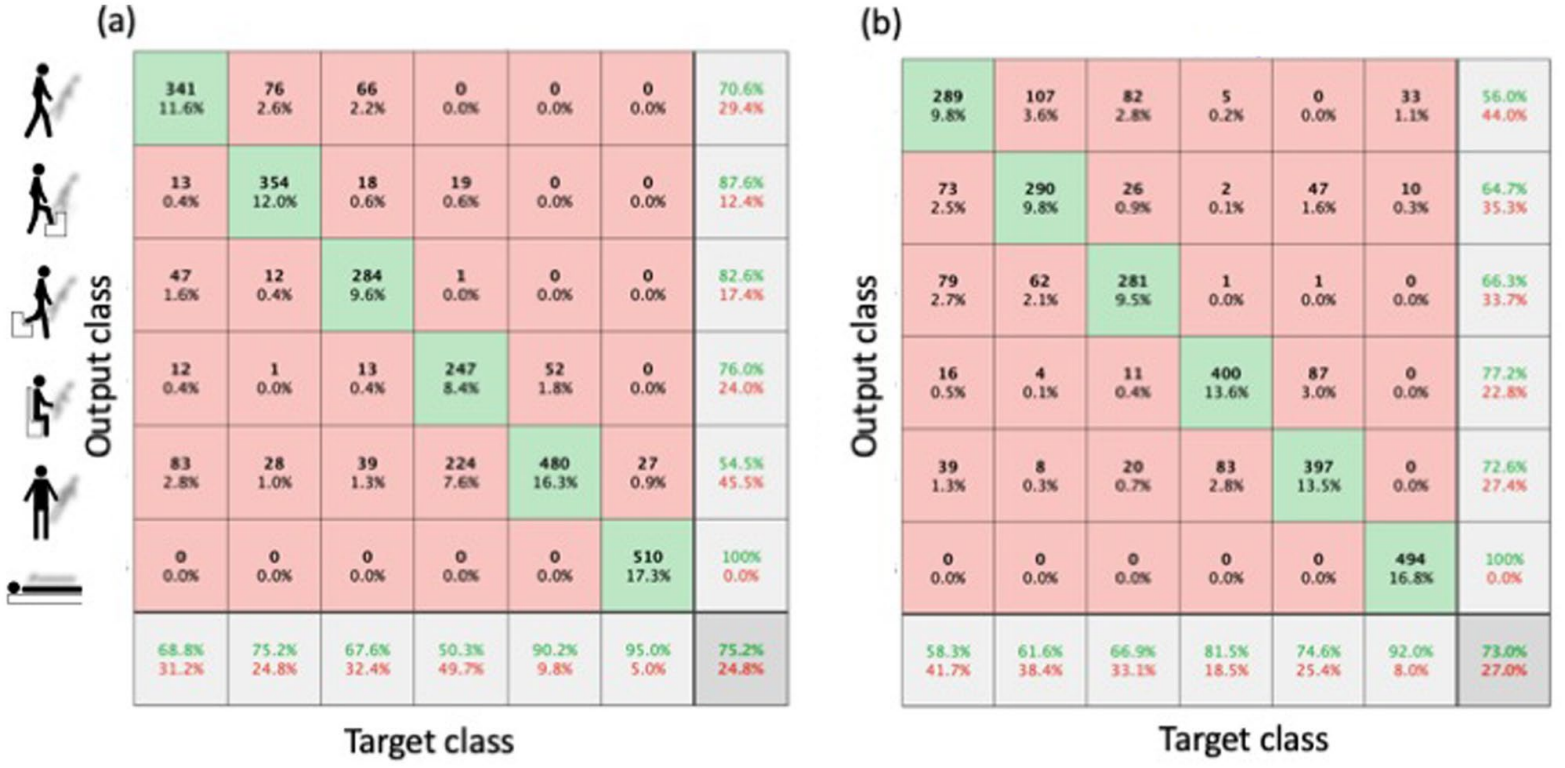
Confusion matrices of human 6-activity classification are compared for 1000-node reservoir. (**a**) Reservoir weight matrix ${W}_{res}$ of small-world network $\left(k,p\right)=\left(4, 0.5\right)$. Accuracy (in green) is 75.2%. (**b**) Conventional sparsely random matrix ${W}_{res}$ with the density of 0.008. Accuracy is 73.0%.

Hereafter, we will introduce the small-world network to the RC and synthesize the reservoir accordingly. We will investigate how the small-world network acts as the reservoir by comparing with a conventional sparsely random weight matrix ${W}_{res}$. We investigate the performance of the RC by processing temporal and serial data. The recognition of images such as handwritten digits and letters are beyond the scope of this study because they require a peculiar preprocessing of image deformation techniques such as reframing and resizing^9^. We implement two benchmark tests; classification of human activity^34^ and time series prediction of Mackey–Glass (MG) chaotic signal^35^. We first study the dependence of the probability of rewiring $p$ on both the performance of the classification of human activity and prediction of the MG chaotic signal shown in Fig. 4a,b, respectively. The human activity includes six motions—walking, walking upstairs, walking downstairs, sitting, standing, and lying down (Fig. 5a). The motions along x-, y-, and z-axes are captured by the accelerator of a smartphone as shown on the bottom of in Fig. 4a^34^. The 1000-node reservoir ${W}_{res}$ is generated for the degree $k=4$. The impact of the degree $k$ on the performance will be discussed later (Fig. 7). In all the benchmark tests, the reservoir network is generated for ten time, and each of ten test runs is conducted by using a different reservoir. The classification accuracy of the human 6-activity improves as $p$ increases from 65.3% at $p=0.0001$ and peaks out to 74.9% at $p=0.5$ (Fig. 4a). While, in the mean square error (MSE) of the MG chaotic signal prediction (Fig. 4b), a 1000-node reservoir ${W}_{res}$ is also generated with the degree $k=4$. The prediction accuracy, which is represented by the MSE is minimized to be $4.98\times {10}^{-6}$ at $p=0.1$. From Fig. 4, it is observed that the sweet spot of the optimum performance of these tests is situated in the range of small-world network, the shaded area of $p=$ 0.01–0.7. The hyperbolic tangent is used for the activation function $f$ in Eq. (1) throughout this study, and sets of parameters such the leaking rate $\alpha$ and input gain $\gamma$ in Eq. (1) and the regularization parameter $\lambda$ in Eq. (3) are tuned for the optimum performance. For the case with the node count $N=1000$, the typical values of the leaking rate $\alpha$, the input gain $\gamma$, and the regularization parameter $\lambda$ are $\left(\alpha , \gamma ,\lambda \right)=(0.3, 1.0, 1.0\times {10}^{-8})$ for the human motion classification and $(0.7, 1.0, 0.2\times {10}^{-8})$ for the MG signal prediction. The results of 2000-node will be discussed later in the manuscript.

We will present the results of the two benchmark tests in details. First, in the human activity classification the captured temporal waveforms along x-, y-, and z-axes for 2.56 s were sampled into 128-sample at a rate of 20 ms as shown on the bottom of Fig. 4a. The data sets of the training and the testing include 7352 and 2947, respectively (see “Methods” section). The accuracy of the human activity classification in the confusion matrix is maximized to 75.2% when $\left(k,p\right)=\left(4, 0.5\right)$ (Fig. 5a), and it is slightly better than that of conventional random weight matrix (73.0%) with the same density of 0.008 as the 1000-node small-world network, which is calculated from Eq. (6) (Fig. 5b).

**Figure 6:**
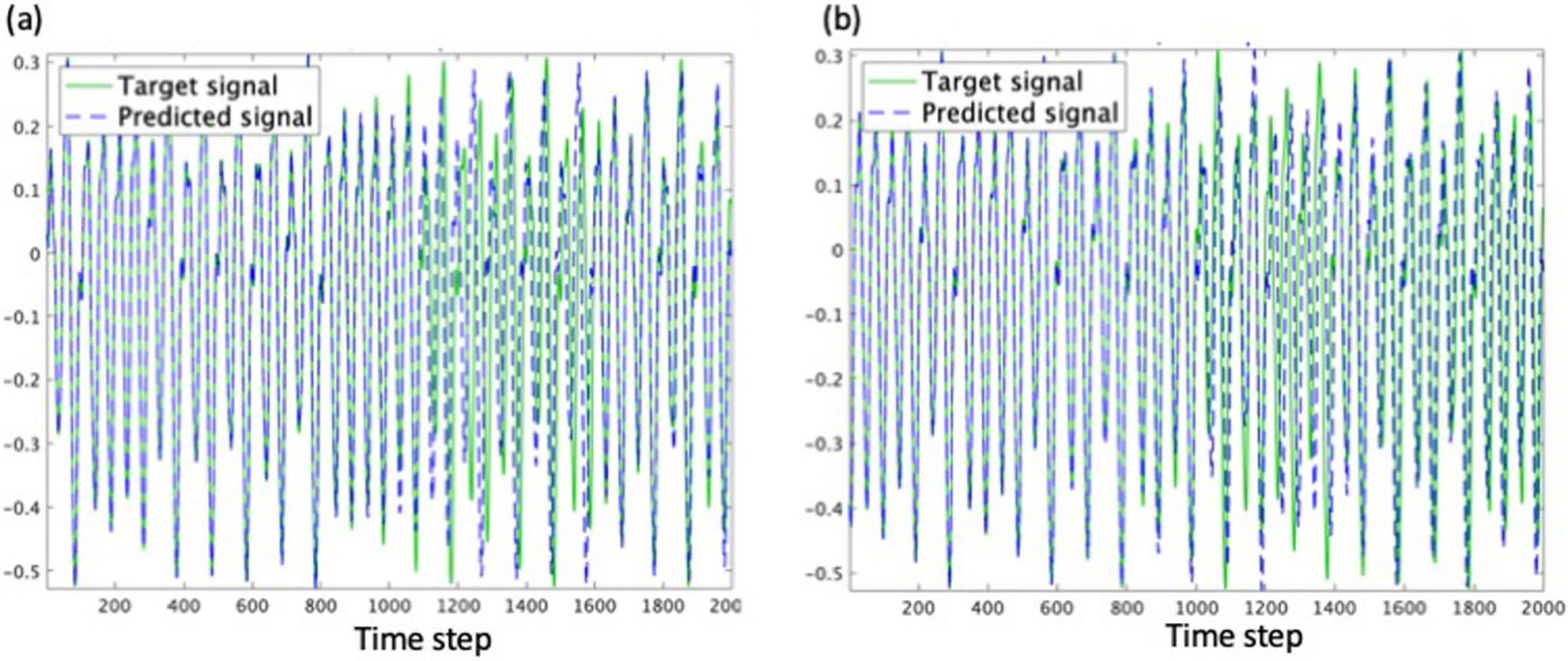
2000 timestep-long waveforms of predicted and that of target MG chaotic time series for 1000-node reservoir. (**a**) Result of reservoir weight matrix ${W}_{res}$ of 1000-node small-world network $\left(k,p\right)=\left(4, 0.1\right)$. Mean square error (MSE) is $2.46\times {10}^{-6}$. (**b**) Conventional sparsely random matrix ${W}_{res}$ with the density of 0.008. MSE is $1.38\times {10}^{-6}$. Results of the two benchmark tests are summarized in Table 1.

In the benchmark test of the MG temporal chaotic signal, a 10,000 timestep-long signal is used. The first 2000 of data are used for the training, and the output weight matrix ${W}_{out}$ is determined by Eq. (3) (see “Methods” section). Then, the next 2000 of the data are used for the prediction. The reservoir consists of 1000-node. The plots of waveforms provide a comparison of the RC results using small-world network as the reservoir with that of sparsely random weight matrix (Fig. 6a,b). The results are summarized in Table 1, including the minimum/maximum mean square errors (MSEs) along with the mean values and the standard deviations for the 10-run. The best MSE of the 1000-node small-world is as low as $2.46\times {10}^{-6}$ (Fig. 6a), slightly larger compared to $1.38\times {10}^{-6}$ of the random weight matrix with the density of 0.008, which is equal to the density of small-world reservoir (Fig. 6b). It can be confirmed that the optimum performance is obtained in the range of small-world as the classification of human activity does. It should be noted that the standard deviation was $1.42\times {10}^{-6}$ which amounts to 28.5% of the MSE value. The impact of the length of training data is examined using the 1000 and the 3000 timestep-long training data, and it is confirmed that the 2000 timestep-long training data is sufficient. For instance, the prediction accuracy was $2.07\times {10}^{-6}$ when the 3000 training data are used, while it is $3.88\times {10}^{-5}$ for the case with the 1000 training data.

**Table 1:** Summary of two benchmark tests using reservoir of small-world and sparsely random ${W}_{res}$ for the cases with $N=$ 1000-node and 2000-node.

	*N* $(k,p)$ Density	Small-world network	Random weight matrix
Max/min accuracy Mean Standard dev (%)	1000 $(\mathrm{4,0.5})$ 0.008	75.2/74.2 74.9 0.25	73.0/71.6 72.8 0.58
	2000 $(\mathrm{4,0.5})$ 0.004	79.2/75.3 77.4 1.80	75.9/73.2 75.4 0.85
Min/max MSE Mean Standard dev	1000 $(\mathrm{4,0.1})$ 0.008	$2.46\times {10}^{-6}$/$7.56\times {10}^{-6}$ $4.98\times {10}^{-6}$ $1.42\times {10}^{-6}$	$1.38\times {10}^{-6}$/$2.22\times {10}^{-6}$ $1.82\times {10}^{-6}$ $3.77\times {10}^{-7}$
	2000 $(\mathrm{4,0.1})$ 0.004	$6.01\times {10}^{-8}$/$6.01\times {10}^{-8}$ $6.01\times {10}^{-8}$ 0	$7.03\times {10}^{-8}$/$1.38\times {10}^{-7}$ $9.00\times {10}^{-8}$ $2.80\times {10}^{-8}$

$N$ number of node, $k$ degree of node*,* $p$ probability of rewiring.

The results of two benchmark tests with the case of the 2000-node are also summarized in Table 1. The confusion matrix of the classification accuracy of human 6-activity and the waveforms of predicted MG chaotic time series are shown in Supple-Figs. 2 and 3, respectively. The performance is considerably improved compared to the results of $N=1000$. The mean value of classification accuracy is increased to 77.4% for the small-world of $\left(k,p\right)=\left(4, 0.5\right)$, and it outperforms the sparsely random weight matrix, whose accuracy is 75.4%. The density of the weight matrix is 0.004, which is equal to the density of small-world reservoir. In the chaotic signal prediction, the mean value of MSE of the small-world reservoir of $\left(k,p\right)=\left(4, 0.1\right)$ is $6.01\times {10}^{-8}$, better than that of random weight matrix, whose mean MSE is $9.00\times {10}^{-8}$.

**Figure 7:**
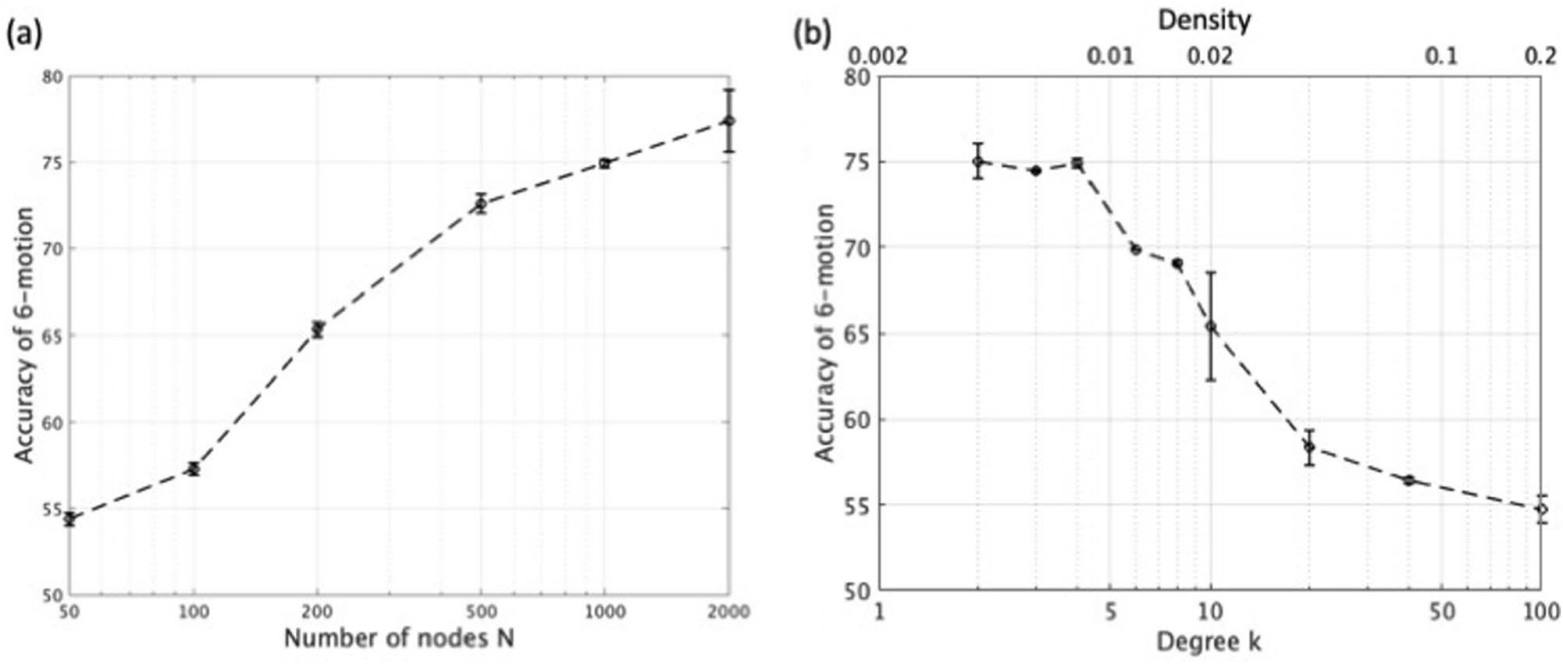
Performance of human activity classification using reservoir weight matrix ${W}_{res}$ generated from the Watts–Strogatz graph. (**a**) Classification accuracy versus the number of nodes $N$. Node degree $k=4$ and the probability of rewiring $p=0.5$ within the range of small-world network are assumed. (**b**) Classification accuracy against the degree of node $k$. $p=0.5$ of 1000-node small-world network is assumed. Horizontal axis on the top is density of matrix ${W}_{res}$ calculated by Eq. (6).

Finally, we investigate the RC performance against the parameters of the small-world network such as the number of nodes $N$ and the degree of node $k$. We will focus on the small-world range $p=$ 0.1–0.7, which is indicated by the shaded area in Fig. 3a, and hence, we assume $\left(k,p\right)=\left(4, 0.5\right)$. First, the dependence of the classification accuracy of the human 6-activity on $N$ is investigated (Fig. 7a). The classification accuracy monotonically improves as the network scales up. The mean value is 54.4% at $N=50$, and it continues to increase to 77.4% at $N=2000$. Next, we will observe the impact of the node degree $k$ on the classification accuracy (Fig. 7b). The accuracy is maximized with relatively small number of degrees around $2\le k\le 4$, and it monotonically degrades as the degree $k$ is increased.

## Discussion

We have conducted two benchmark tests—the classification of human activity and the time series prediction of the Mackey–Glass chaotic system. It has been demonstrated that the optimum performance is obtained from the reservoir in the range of small-world network bounded by $p=$0.01–0.7 and $k<20$. Based on this observation, a guiding principle has been presented to systematically synthesize a reservoir of RC by exploring the small-world network nature of highly-clustered with the short characteristic path length. We expect that this study will draw attention to the versatile capability of the small-world network in the research of neural networks and help understand architectures of the RC in-depth.

## Methods

### Generation of weight matrix of reservoir

**Figure 8:**
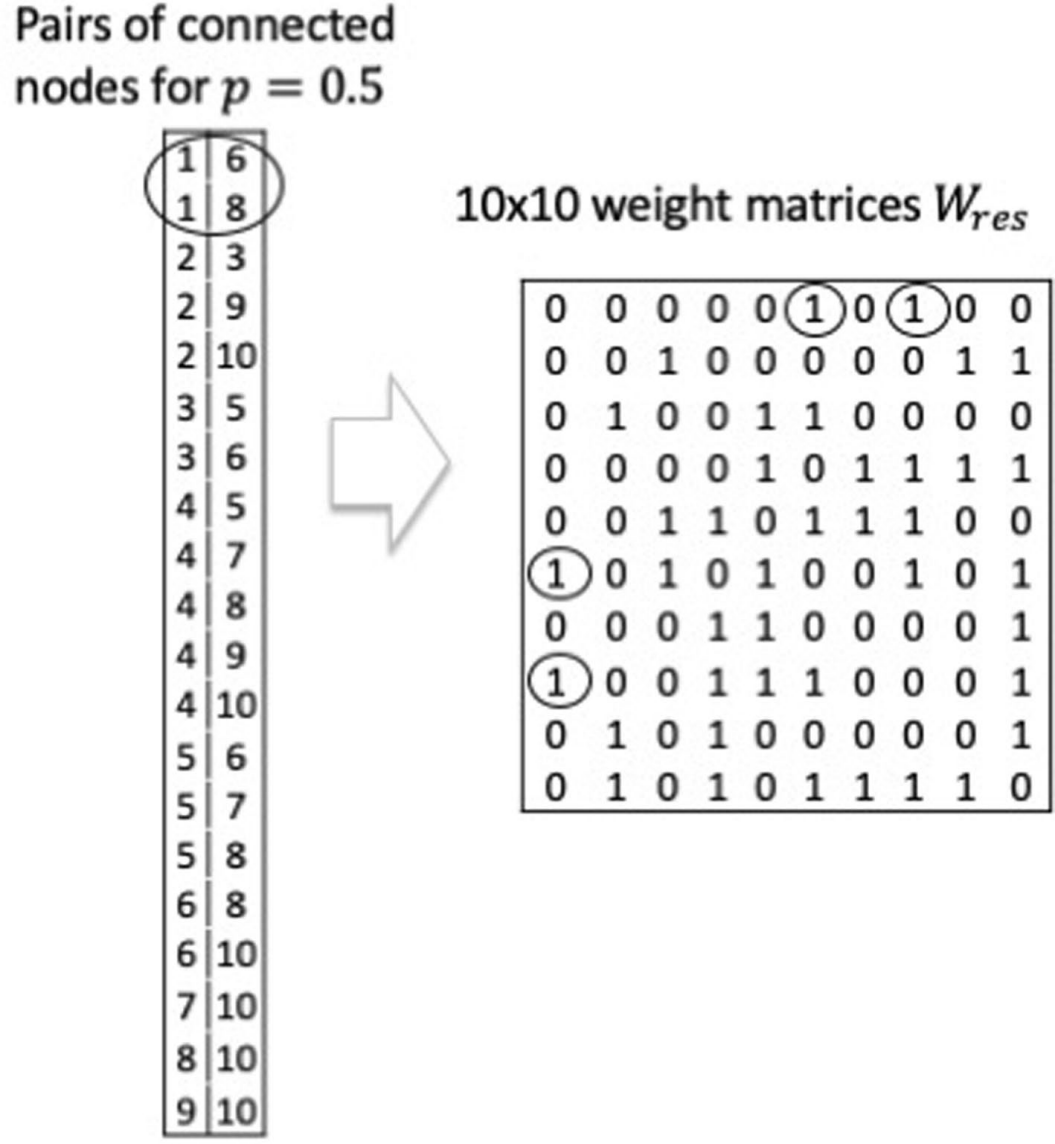
Method for generating the weight matrix ${W}_{res}$ of the reservoir of 10-node ($N=10$) networks with $\left(k,p\right)=(2, 0.5)$. Table of pairs of connected nodes on the l.h.s. and $10\times 10$ weight matrix ${W}_{res}$. For instance, connections of node 1, pairs of nodes, $(1, 6)$ and $(1, 8)$ reflect on the weight matrix ${W}_{res}$, as marked by circles.

The method of generating the weight matrix ${W}_{res}$ of the reservoir of 10-node ($N=10$) network with the node degree $k=2$ is illustrated (Fig. 8). The table on the l.h.s. represents the pairs of connected nodes for $p=0.5$. From this table, the weight matrix ${W}_{res}$ of the reservoir for the bidirectional connection can be generated, as shown on the r.h.s. Throughout the simulation, the nonzero elements of ${W}_{res}$ take a binary value, and it is assumed that all the connections are bidirectional, resulting in a symmetric weight matrix. Although, the nonzero elements can take an arbitrary positive value, and the asymmetric matrix may be another option.

### Generation algorithm of Watts–Strogatz graph^12^

Creating the Watts–Strogatz graph through two basic steps:

1. Create a ring lattice with $N$-node of the mean degree 2 $k$ (on the l.h.s. on the top of Fig. 2). Each node is connected to its nearest neighboring 2 $k$ nodes.
2. For each edge or link in the graph, rewire the target node with probability $p$. The rewired edge cannot be a duplicate or self-loop. This results in a partially ($p=0.5$) or totally disordered ($p=1$) topologies (on the r.h.s. on the top and at the bottom of Fig. 2, respectively).

The basic Matlab code is available at: https://jp.mathworks.com/help/matlab/math/build-watts-strogatz-small-world-graph-model.html?lang=en. The clustering coefficient $C(p)$ is calculated using Eqs. (4) and (5) by following the aglgorithm^33^. The Matlab code of $C(p)$ is available at https://github.com/mdhumphries/SmallWorldNess.

### Classification of human activity^34^

The data set of the human activity is available at http://archive.ics.uci.edu/ml/machine-learning-databases/00240/UCI HAR Dataset.zip.

It includes six motions—walking, walking upstairs, walking downstairs, sitting, standing, and laying down. The captured temporal waveforms along x-, y-, and z-axes for 2.56 s were sampled into 128-sample at the rate of 20 ms. The data sets of the training and the testing are 7352 and 2947, respectively. For the training and classification of the human activity in RC (Fig. 1), the dimensions of weight matrices ${W}_{in}$, ${W}_{res}$, and ${W}_{out}$ of Eqs. (1), (2) and (3) are $L=1, M=6$ and $N$ is specified, for instance 1000 etc.

### Time series prediction of Mackey–Glass (MG) chaotic signal^35^

The time series data and the basic Matlab code are available at:

A minimalistic sparse Echo State Networks demo with Mackey–Glass (delay 17) data in "plain" Matlab/Octave from https://mantas.info/code/simple_esn (c) 2012–2020 Mantas Lukosevicius.

Distributed under MIT license https://opensource.org/licenses/MIT.

For the training and prediction of MG chaotic time series, the dimensions of weight matrices ${W}_{in}$, ${W}_{res}$, and ${W}_{out}$ of Eqs. (1), (2) and (3) are $L=1, M=1$ and $N$ is specified, for instance 1000 etc.

## Supplementary Information

Supplementary Information.

## Data Availability

The raw data sets used in the simulations are available (see “Methods”).

## Code availability

The Matlab-based codes used for simulations are partly available (see “Methods”).
