# Supplementary material for: Guiding principle of reservoir computing based on “small-world” network
Source: Sci Rep. 2022 Oct 6;12:16697. doi: 10.1038/s41598-022-21235-y (PMC9537422; doi:10.1038/s41598-022-21235-y)
Supplement: Supplementary file 1 — Supplementary Information. [file 41598_2022_21235_MOESM1_ESM.pdf]

# Guiding Principle of Reservoir Computing based upon "Small-world" Network

Ken-ichi Kitayama*

National Institute of Information and Communications Technology, Tokyo, 184-8795, Japan.
Hamamatsu Photonics K.K., Hamamatsu, 434-8601, Japan

* Corresponding author. email: kitayama@ieee.org

**Additional information**
**Supplementary information**

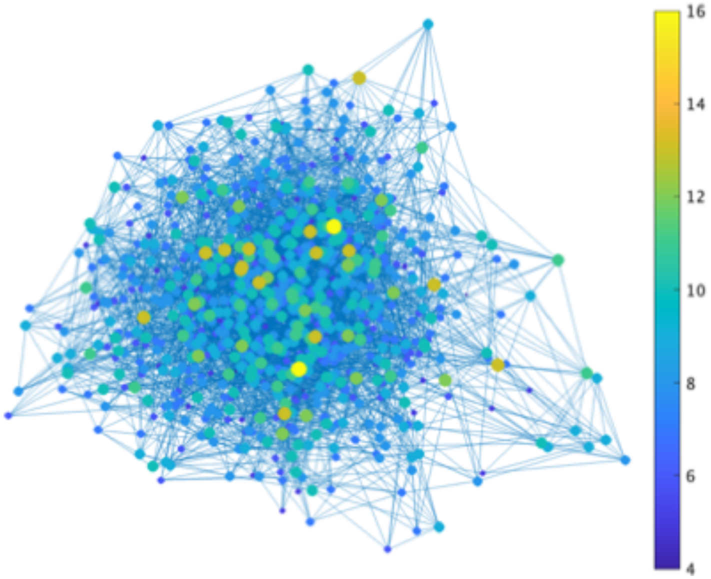


**Suppl-fig.1** 1000-node network with $(k, p) = (4, 0.5)$. There are several clustering hubs, which connect with up to 14~16 nodes are indicated in orange and yellow of the color bar.

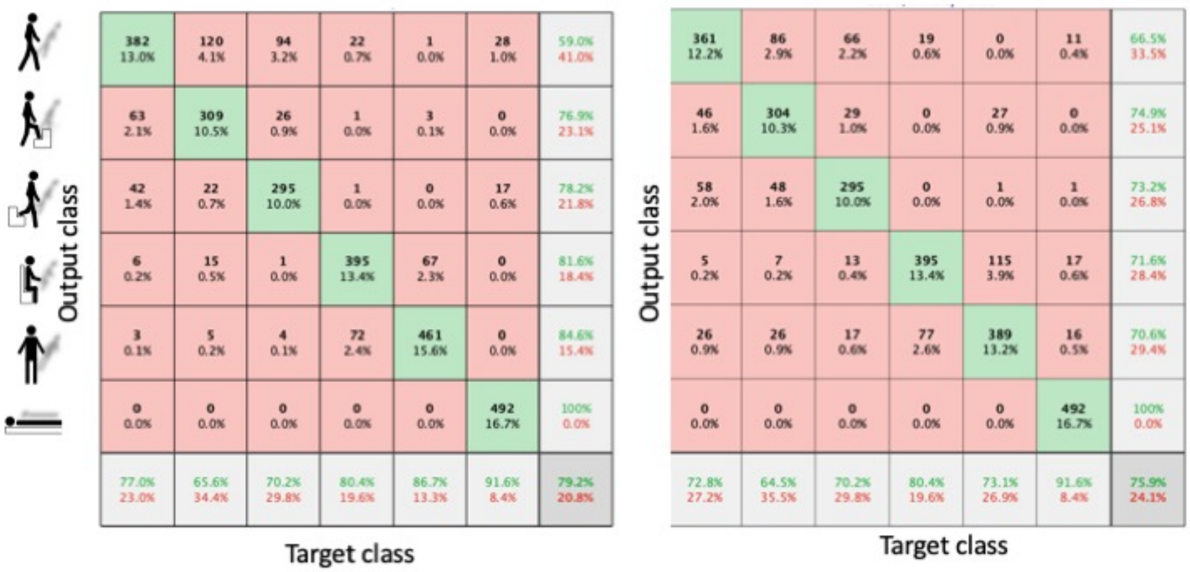


**Suppl-fig.2** Confusion matrices of human 6-activity classification are compared for 2000-node reservoir. **a** Reservoir weight matrix $W_{res}$ of small-world network $(k, p) = (4, 0.5)$. The accuracy (in green) is 79.2%. **b** Conventional sparsely random matrix $W_{res}$ with the density of 0.004. The accuracy is 75.9%.

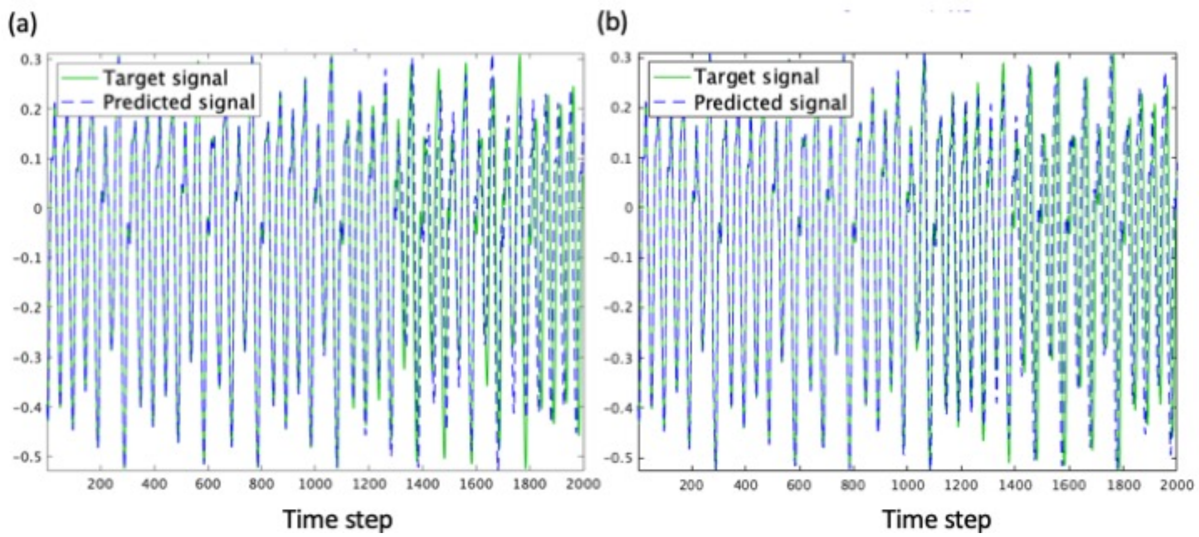


**Suppl-fig.3** 2000 timestep-long waveforms of predicted and that of target MG chaotic time series for 2000-node reservoir. **a** Result of reservoir weight matrix $W_{res}$ of 2000-node small-world network $(k, p) = (4, 0.1)$. Mean square error (MSE) is $6.01 \times 10^{-8}$**.** **b** Conventional sparsely random matrix $W_{res}$ with the density of 0.004. MSE is $7.03 \times 10^{-8}$**.** Results of the two benchmark tests are summarized in Table 1.
